# Study of Lethal Congenital Malformations at a Tertiary-Care Referral Centre in North India

**DOI:** 10.7759/cureus.7502

**Published:** 2020-04-01

**Authors:** Purnima Tiwari, Madhavi M Gupta

**Affiliations:** 1 Obstetrics and Gynaecology, All India Institute of Medical Sciences, Bhopal, IND; 2 Obstetrics and Gynaecology, Maulana Azad Medical College, New Delhi, IND

**Keywords:** congenital birth defect, stillbirth, neonatal mortality

## Abstract

Lethal congenital malformations (LCMs) are fatal birth defects that are an important cause of fetal/neonatal death. There is a lack of informative data about these malformations in India, a country that shares the maximum burden of neonatal mortality due to congenital birth defects. Therefore, we conducted a retrospective analysis to know the prevalence of LCMs in late pregnancy, to find out associated factor/variables and to evaluate fetal/neonatal outcome of such anomalies; at a tertiary-care referral centre in North India. All deliveries with LCMs after 24 weeks of gestation were included in the study. Data about antepartum history (maternal age, parity, education, socioeconomic status, consanguineous marriage, folic acid intake, any chronic medical disorder, availability of anomaly scan, unplanned pregnancy); intrapartum events (gestational age at delivery, mode of delivery); postpartum events (weight of the baby, gender of the baby); newborn evaluation; and details of hospital stay were recorded from medical record sheet over the duration of one year. We found that anencephaly, severe meningomyelocele, multicystic dysplastic kidneys and non-immune hydrops with major cardiac defects were more prevalent among all LCMs. On the evaluation of the various studied variables, maximum babies with LCMs were born to mothers who were between 20 and 35 years of age, those who were illiterate, belonged to middle/lower socio-economic class, multigravida, and those who had no detailed anomaly scan. We feel that there is an urgent need to formulate a universally accepted definition of LCMs, to identify preventable risk factors and to formulate management strategy for both mother and liveborn baby with LCMs, in order to minimize the hidden burden of these defects in stillbirth/ perinatal/ neonatal mortality statistics.

## Introduction

Congenital anomalies are one of the major causes of neonatal mortality and morbidity worldwide. Serious birth defects account for 7% of overall neonatal mortality and 94% of these births occur in middle and low-income countries [1]. India contributes a significant proportion (28%) in global burden of neonatal mortality due to congenital birth defects [2]. Also, congenital malformations account for 8% to 15% perinatal mortality and 15% to 16% neonatal mortality in India [3]. Though not all congenital malformations lead to the death of the fetus, some malformations are proven fatal either in utero or in early neonatal life. These lethal congenital malformations (LCMs) are the ones that are the most hidden and most neglected part of stillbirth/ perinatal/ neonatal mortality statistics, as these are generally not amenable to treatment.

Although there is abundant data present on congenital malformations in India, there is a lack of studies solely focusing on LCMs. There is an urgent need for accurate quantification of the burden of LCMs among all congenital anomalies in the population of the country in order to improvise targeted preventive measures and management options for these malformations. LCMs diagnosed in late fetal life still pose a management dilemma for healthcare professionals regarding the fetus as well as the mother. Therefore this issue should be addressed by policymakers in the country for the reduction of neonatal mortality.

The main objectives of this study were to know the prevalence of LCMs in late pregnancies, to predict variables for such malformations and to evaluate the fetal outcome of such lethal anomalies till the neonatal period.

## Materials and methods

This was a retrospective study based on hospital records conducted at a tertiary-care referral centre in North India. Data from hospital birth registry of all deliveries occurred after 24 weeks of gestation between January 2014 and December 2014 were studied to identify deliveries with LCMs, and such records were then reviewed to look for antenatal history of mother, intrapartum and postpartum events, evaluation of newborn, and details of hospital stay of the newborn. All deliveries with LCMs which occurred during the given period after 24 weeks of gestation were included for analysis. In our institute, all the delivered babies are screened for LCMs, and if suspected, these malformations are confirmed by necessary investigations like X-ray, ultrasound examination, karyotyping of baby, and echocardiography.

We considered those malformations as LCMs which were identified and listed as lethal by Wilkinson *et al*. [4]. We retrieved the data about various factors which included mother’s age, mother’s parity, mother’s education, mother’s socioeconomic status, consanguineous marriage, planned/unplanned pregnancy, availability of anomaly scan, periconceptional folic acid intake, previous baby with a congenital anomaly, history of chronic medical disorders, mode of delivery, gestational age at delivery, birthweight, and gender of the baby.

Mother’s socioeconomic status was assessed using a modified Kuppuswamy’s scale [5]. For all the LCM cases, the availability of a detailed second-trimester anomaly scan was looked for. The chronic medical disorders which we specifically enquired in the study included diabetes mellitus (excluding gestational diabetes), hypertensive disorders, heart diseases, autoimmune disorders, and renal disorders. The prevalence of each lethal malformation in late gestation was recorded. Babies born alive with lethal malformations were kept on supportive palliative care. Survival period for all these newborns was noted, and neonatal outcome was assessed by calculating the average survival period for all the malformations.

Data were analysed, and the prevalence of various LCMs per 10000 births was calculated. Percentages and total numbers were calculated for each of the studied variables in those LCM cases. Percentages of liveborn and stillborn babies were calculated, and among the liveborn babies, mean was calculated for the survival hours.

## Results

Over the one year of the study period, a total of 14,530 deliveries and 14,681 births occurred after 24 weeks of gestation. The prevalence of all congenital anomalies was 256.11 per 10,000 births (2.56%). In all, 76 babies were born with LCMs, and so, the prevalence of LCMs overall was 51.76 per 10,000 births (0.52%).

Anencephaly (12.94 per 10,000 births), severe meningomyelocele (7.49 per 10,000 births), multicystic dysplastic kidneys (6.13 per 10,000 births), and non-immune hydrops with major cardiac defects (5.44 per 10,000 births) were more prevalent among all LCMs. We had four cases of sirenomelia and two cases of alobar holoprosencephaly, which are relatively rare disorders (Table 1) [6-13].

**Table 1:** Prevalence of various LCMs found in our study and their comparison with previously reported results LCM, lethal congenital malformations [6-13]

S. No.	Malformation	Total number of children born	Prevalence (per 10,000 births)	Reported
1.	Anencephaly	19	12.94	7.33/1000 births
2.	Trisomy 13	5	3.41	1 in 7,906 births
3.	Trisomy 18	3	2.04	1 in 3,762 births
4.	Holoprosencephaly (Alobar)	2	1.36	1.3/10,000 births (overall)
5.	Hypoplastic left heart syndrome	1	0.68	1 in 4,344 births
6.	Multicystic dysplastic kidney	9	6.13	1 in 4400 births
7.	Congenital severe hydrocephalus	5	3.41	0.69/1000 births
8.	Sironemelia	4	2.72	0.8-1 case/100,000 births
9.	Severe meningomyelocele	11	7.49	1.74/1000 births
10.	Large encephalocele	4	2.72	1 in 12,235
11.	Giant omphalocele	1	0.68	1 in 5386 (overall)
12.	Non-immune hydrops with major cardiac defects	8	5.44	0.65/10,000 births
13.	Congenital severe diaphragmatic hernia	3	2.04	1 in 2000-5000 live births
14.	Renal agenesis (bilateral)	1	0.68	1/10,000 births

Apart from the above listed LCMs, we also had one case of lissencephaly and one case of multiple intestinal atresia. All newborns with LCMs were singleton issues.

Upon evaluation of the various studied variables, maximum babies with LCMs were born to mothers who were between 20 and 35 years of age (72.37%), those who were illiterate (51.32%), belonged to middle/lower socio-economic class (77.63%), multigravida (59.21%) and those who had no detailed anomaly scan (63.16%). Consanguineous marriage was present only in eight cases. Only 14 pregnancies were planned, and out of these, only 10 mothers took periconceptional folic acid. The mothers with a history of chronic medical disorders contributed 18.42% of all the LCM cases. Nineteen mothers (25%) had a history of a previous baby born with congenital anomalies. A substantial proportion of the LCM babies were born before 34 weeks’ of gestation (43.42%), and majority of them had birth weight between 1 and 2.5 kg (75%). The total number of male babies (52.63%) was more than female babies (47.37%). Five babies were born from the lower segment cesarean section (LSCS), and 71 mothers had vaginal deliveries (Table 2).

**Table 2:** Study of various factors in mothers who delivered LCM babies LCM, lethal congenital malformations; LSCS, lower segment caesarean section

S. No.	Factor	Number of mothers	Percentage
1.	Maternal age		
	<20 years	2	2.63%
	20-35 years	55	72.37%
	>35 years	19	25%
2.	Maternal education		
	Literate	37	48.68%
	Illiterate	39	51.32%
3.	Socioeconomic status		
	Upper	17	22.37%
	Middle/Lower	59	77.63%
4.	Consanguineous marriage		
	Yes	8	10.53%
	No	68	89.47%
5.	Mother’s parity		
	Primigravida	31	40.79%
	Multigravida	45	59.21%
6.	Unplanned pregnancy		
	Yes	62	81.58%
	No	14	18.42%
7.	Periconceptional folic acid intake		
	Yes	10	13.16%
	No	66	86.84%
8.	Availability of anomaly scan		
	Yes	28	36.84%
	No	48	63.16%
9.	Previous baby with congenital anomaly		
	No	57	75%
	Yes	19	25%
10.	Chronic medical disorder in mother		
	No	62	81.58%
	Yes	14	18.42%
11.	Gestational age		
	28-34 weeks	33	43.42%
	34- 37 weeks	29	38.16%
	>37 weeks	14	18.42%
12.	Birth weight		
	<1 kg	7	9.21%
	1-2.5 kg	57	75%
	>2.5 kg	12	15.79%
13.	Sex of baby		
	Female	36	47.37%
	Male	40	52.63%
14.	Mode of delivery		
	Vaginal	71	93.42%
	LSCS	5	6.58%

LSCS was mainly done for the maternal indication: two for obstructed labor, one for ruptured uterus, and two for severe antepartum hemorrhage. A total of 28 babies (36.84%) were liveborn, while 48 (63.16%) were stillborn. Those who were liveborn had a mean survival time of 14 hours. All liveborn babies had a 100% mortality rate in the neonatal period.

## Discussion

LCMs are the most hidden and most neglected part of stillbirth/ perinatal/ neonatal mortality statistics. Due to the lack of adequate studies about LCMs from India, dilemma exists regarding their exact prevalence, about factors associated with these malformations and their fetal/neonatal outcome. We, therefore, conducted this study to know the prevalence of LCMs in late pregnancies, to predict variables for such malformations and to evaluate outcomes of such lethal anomalies till the neonatal period. To the best of our knowledge, this is first of its kind of study solely evaluating LCMs in India, a country that contributes significantly to the burden of babies born with congenital anomalies each year.

The nationwide prevalence of congenital malformations in India is 70/10,000 births [6]. In our study, we found it to be 256.11/10,000 births, quite higher than previously reported. Data on the prevalence of overall LCMs is still sparse in the country. In a retrospective study, Bai *et al*. evaluated 13,964 consecutive births over one year and reported that 0.34% of babies were born with LCMs [14]. Almost similar to the above-mentioned result, we had 0.52% of babies born with LCMs. The proportion of stillbirths (64% vs. 63.16%, respectively) and livebirths (36% vs. 36.84%, respectively) with LCMs was also comparable in both the studies.

There is a lot of variation in the reported incidences of various LCMs from different parts of the country. The difference in the definition of a particular anomaly to call it as a lethal one and differences in easy availability of screening/diagnostic modality may be the possible reasons behind this heterogeneity. In our study, LCMs with higher prevalence than previously reported, in the country or worldwide, included trisomy 13, holoprosencephaly, multicystic dysplastic kidneys, sirenomelia, large encephalocele, non-immune hydrops with major cardiac defects, and congenital severe diaphragmatic hernia [7-12]. Some LCMs were found to be less prevalent than previously reported like anencephaly, hypoplastic left heart syndrome, severe meningomyelocele, giant omphalocele, and bilateral renal agenesis [6-7,13]. We could not find the exact prevalence of some anomalies in literature, for example, congenital severe hydrocephalus with absent or minimum brain growth. Therefore, we compared it with the overall prevalence of congenital hydrocephalus. A similar situation was encountered for congenital severe diaphragmatic hernia with hypoplastic lungs, severe meningomyelocele, large encephalocele, and alobar holoprosencephaly. Since no universally accepted definition and list of lethal anomalies exist in literature to date, we emphasize the need for more studies about LCMs. The term "lethality" needs to be described more descriptively and precise information about such anomalies will solve the mystery regarding exactly which anomalies to be called as lethal ones.

Since there is a lack of studies in the literature evaluating factors particularly for LCMs, we compared our results with other studies that evaluated all the congenital malformations. Similar to our result, Sarkar *et al*. found that congenital anomalies were more common in the maternal age group of 21-30 years and multiparas [15]. Although consanguineous marriages have been predicted as a risk factor for congenital anomaly, maximum babies with LCMs were born out of non-consanguineous marriage in our study. The difference in the result may be due to differences in the ethics and cultural practices of the population of the catchment area, where consanguineous marriage is not much prevalent. A higher risk of certain congenital malformations was reported to be found in parents belonging to a lower class of socioeconomic strata and with lower maternal education level [16]. Similarly, we also found a slightly higher proportion of LCMs in illiterate mothers and significantly higher in mothers belonging to the lower/middle socioeconomic class.

Majority of the babies with LCMs were born as a result of unplanned pregnancies. Most of such mothers neither took periconceptional folic acid nor had a detailed anomaly scan in the second trimester. The association between lack of folic acid and congenital anomalies has been already described in the literature [17]. These findings stress upon the need for contraceptive counseling of reproductive age women, preconception counseling in high-risk patients, good antenatal care to detect such anomalies in time, and timely intervention to reduce the burden of these anomalies in late pregnancies. Though the history of chronic medical disorders in mother and history of a previous baby affected with congenital anomaly did not seem to affect the outcome in current pregnancy regarding LCMs, future prospective studies are needed to find their association with LCMs.

In our study, the proportion of male babies with LCMs (52.63%) was slightly higher than that of female babies. Similar to our results, a previous study from India reported that male babies with congenital malformations (56%) outnumbered female babies (41%) [18]. We found that maximum babies were born between 28 and 34 weeks of gestation and had birth weight between 1 and 2.5 kg. A total of seven babies (9.21%) had a birth weight below 1 kg. Similarly, Bai *et al*. found that in total, 8% of babies with LCMs had a birth weight below 1000 g [14]. Though we provided supportive care at our best to liveborn babies, no baby could survive more than one month of life and there was 100% mortality. Similar to our results, Bai *et al*. also reported 100% mortality of the babies born with LCMs [14].

Our study had some limitations as well which should be known while interpreting the results. Firstly, it was a retrospective study; therefore, we had to rely on the history recorded in medical records. Secondly, the definition of LCMs is still not well described in the literature. Also, the study was conducted at a tertiary care hospital. This imparts a selection bias, and hence, it may be difficult to generalize the results outside the study settings. Furthermore, it was a single-centre study, and so whether the results represent the true prevalence of LCMs in the population as a whole remains doubtful. However, as our institute receives patients from all parts of the country, we can assume sufficient heterogeneity in the study sample to represent the general population. Moreover, we believe that by providing insight about LCMs, which are the hidden burden of perinatal and neonatal mortality, this study will pave the path for further prospective studies on this subject in the country.

## Conclusions

The prevalence of babies born with LCMs was 51.76 per 10,000 births (0.52%) with 100% mortality either in-utero or in early neonatal life. The majority of such babies were the results of unplanned pregnancies and were born to mothers aged between 20 and 35 years, illiterate, belonging to middle/lower socio-economic class, multigravida and those who had no detailed anomaly scan. Good antenatal care can help in reducing these malformations in late pregnancy by timely detection and appropriate intervention. There should be more focus on pre-conceptional counseling. Moreover, these lethal malformations should be reported at pan-country level and a proper database about LCMs should be established, which is lacking at present. Moreover, when these malformations are detected in late gestation, considering 100% mortality of the babies, proper counseling of the mother should be done. We recommend future multi-centre studies on this aspect encompassing multiple plausible variables affecting LCMs, so as to more comprehensively evaluate the preventable risk factors associated with such lethal malformations.
